# Approaches to improving the contribution of the nursing and midwifery workforce to increasing universal access to primary health care for vulnerable populations: a systematic review

**DOI:** 10.1186/s12960-015-0096-1

**Published:** 2015-12-18

**Authors:** A. J. Dawson, A. M. Nkowane, A. Whelan

**Affiliations:** Faculty of Health, University of Technology Sydney (UTS) World Health Organization Collaborating Centre for Nursing, Midwifery and Health Development, Jones Street, Sydney, NSW Australia; Health Workforce Department, World Health Organization, Geneva, Switzerland; University of New South Wales (UNSW), Sydney, Australia; Sydney Local Health District, Sydney, NSW Australia

**Keywords:** Nursing, Midwifery, Access to health care, Primary health care, Vulnerable populations

## Abstract

**Background:**

Despite considerable evidence showing the importance of the nursing and midwifery workforce, there are no systematic reviews outlining how these cadres are best supported to provide universal access and reduce health care disparities at the primary health care (PHC) level. This review aims to identify nursing and midwifery policy, staffing, education and training interventions, collaborative efforts and strategies that have improved the quantity, quality and relevance of the nursing and midwifery workforce leading to health improvements for vulnerable populations.

**Methods:**

We undertook a structured search of bibliographic databases for peer-reviewed research literature using a focused review question and inclusion/exclusion criteria. The quality of retrieved papers was appraised using standard tools. The characteristics of screened papers were described, and a deductive qualitative content analysis methodology was applied to analyse the interventions and findings of included studies using a conceptual framework.

**Results:**

Thirty-six papers were included in the review, the majority (25) from high-income countries and nursing settings (32). Eleven papers defined leadership and governance approaches that had impacted upon the health outcomes of disadvantaged groups including policies at the national and state level that had led to an increased supply and coverage of nursing and midwifery staff and scope of practice. Twenty-seven papers outlined human resource management strategies to support the expansion of nurse’s and midwives’ roles that often involved task shifting and task sharing. These included approaches to managing staffing supply, distribution and skills mix; workloads; supervision; performance management; and remuneration, financial incentives and staffing costs. Education and training activities were described in 14 papers to assist nurses and midwives to perform new or expanded roles and prepare nurses for inclusive practice. This review identified collaboration between nurses and midwives and other health providers and organizations, across sectors, and with communities and individuals that resulted in improved health care and outcomes.

**Conclusions:**

The findings of this review confirm the importance of a conceptual framework for understanding and planning leadership and governance approaches, management strategies and collaboration and education and training efforts to scale up and support nurses and midwives in existing or expanded roles to improve access to PHC for vulnerable populations.

## Background

Universal access is an important step towards universal health coverage to ensure that all people receive the health services they need without suffering financial hardship [1]. Evidence suggests that the provision of affordable, acceptable, high-quality health services [2] leads to better access to necessary care and improved population health, particularly for growing numbers of vulnerable individuals and communities [3] who experience significant health disparities [4]. However, achieving universal access to reduce health inequity and realize improved health outcomes requires competent and motivated nurses and midwives [5] who form the largest group of the world’s health workforce.

The coverage of nurses and midwives, in terms of adequate numbers and their appropriate distribution in locations where the community can access them, is critical [6]. A direct association has been observed between health worker density and maternal, infant and child survival. In particular, the density of nurses has been found to have a significant independent effect on maternal mortality which has not been demonstrated for doctors [7]. Recent calculations also show that scaling up midwifery-delivered interventions from present baseline levels in 78 countries could significantly reduce maternal deaths, stillbirths and neonatal deaths [8].

These findings reveal the importance of accessible nursing and midwifery-led health care within the community at the primary health care level [9, 10]. Nurses and midwives can play a key role in empowering patients and strengthening community involvement in their health [11] through knowing and understanding the health needs of local populations and targeting interventions to meet the wider determinants of health [12]. The education and training, as well as the socio-culturally diverse composition of the nursing and midwifery workforce, may facilitate the appropriate delivery of relevant health care [13, 14] and promote health equity among distinct populations [15, 16].

In addition to adequate numbers of competent nurses and midwives whose backgrounds reflect the diversity of the population they serve, effective human resources for health leadership and management is also an important consideration in the delivery of accessible health care. Well-regulated nursing staff with the appropriate workloads and skills mix has been associated with improved patient satisfaction and health outcomes [17, 18], while inadequate nurse staffing levels have been associated with an increase in adverse events and poor care [19]. However, nurses and midwives cannot deliver care on their own, and in order to achieve universal access and health equity, partnership and collaboration are necessary at the policy level [20] and in practice [21].

Despite considerable evidence showing the importance of the nursing and midwifery workforce, there are no systematic reviews outlining the contribution that these cadres have made to achieving universal access and reducing health care disparities at the primary health care level. Research gaps have been noted in this broad area [4]. Insight into how nurses and midwives can be best placed and supported to deliver care to address the health needs of vulnerable groups at the primary health care (PHC) level, or the first level of contact, should inform the decisions of policy makers with respect to realizing efficient workforce and service planning to achieve health equity. This paper aims to identify nursing and midwifery policy, staffing, education and training interventions and collaborative efforts and strategies that have been found to improve the quantity, quality and relevance of the nursing and midwifery workforce that have ultimately led to health improvements. In particular, the review sought to identify nursing and midwifery workforce interventions and approaches that have led to:

- An increase in the number of well-trained, motivated nurses and midwives to provide the services to meet patients’ needs based on the best available evidence
- The provision of greater access to nursing and midwifery health services, care, information and essential medicines and technologies to diagnose and treat medical problems and
- A reduction in the cost of care making nursing and midwifery health services more affordable to those suffering financial hardship

### A framework for understanding the factors contributing to universal access

**Fig. 1:**
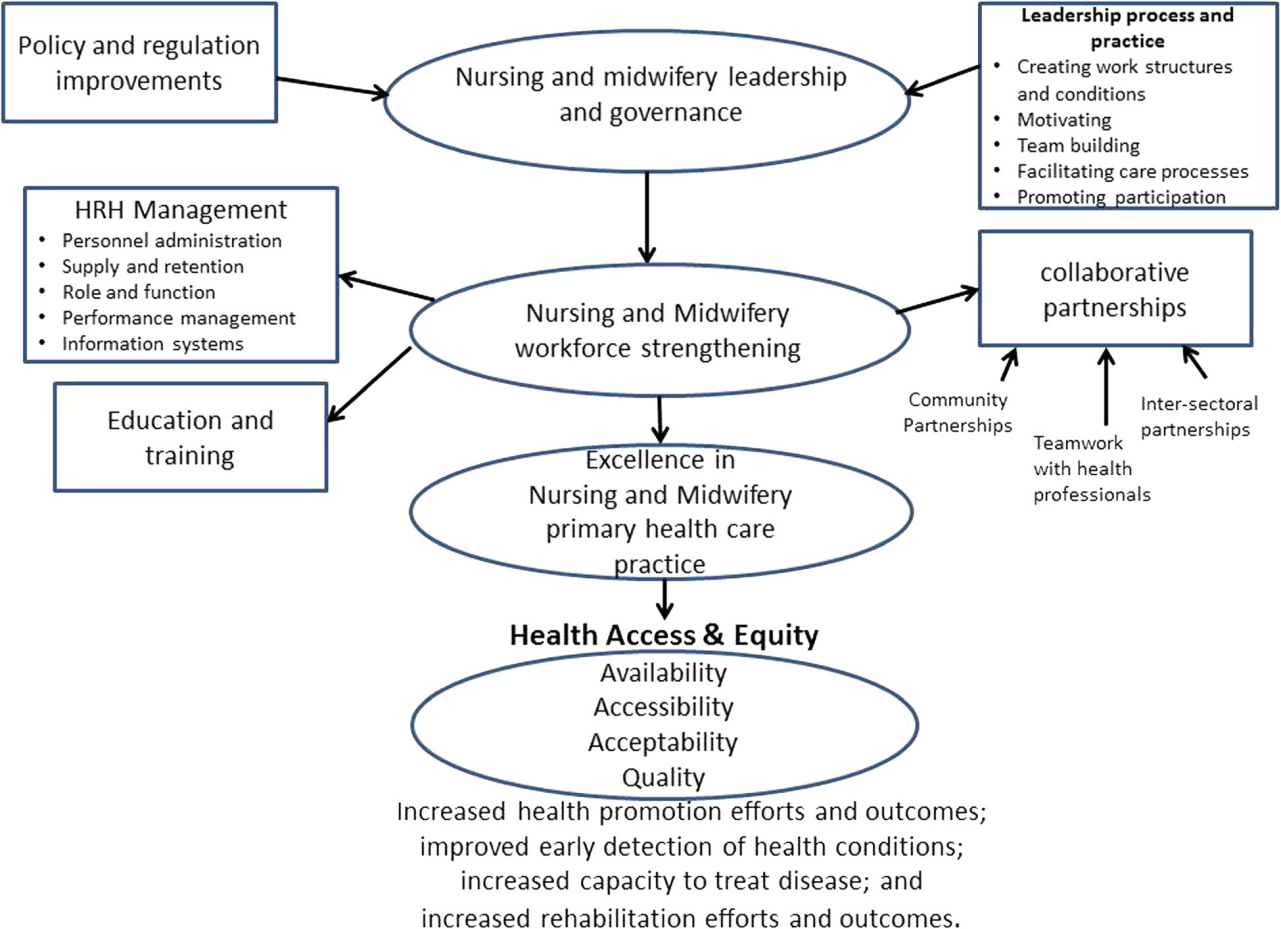
A conceptual framework for examining nursing and midwifery leadership and workforce interventions and their impact on universal health access

A framework was developed to conceptualize the various elements under investigation and the relationships between them (see Fig. 1). This framework is based upon the approach outlined in a discussion paper prepared for the 2014 WHO Global Forum for Government Chief Nursing and Midwifery Officers [22]. The framework helps to describe the link between nursing and midwifery leadership and governance, workforce strengthening interventions, nursing and midwifery practice and universal access and health equity. The link between leadership and governance on action and intervention is drawn from evidence of the impact of leadership upon patient outcomes [23] and acknowledges the importance of leadership at the management level [24] and the practice level in service delivery [25, 26]. Nursing and midwifery workforce areas are based upon human resources for health (HRH) performance fields that can describe both interventions and indicators to support and assess HRH performance [27].

## Methods

Eight bibliographic databases, Google Scholar and the reference lists of key papers were systematically searched to retrieve research literature. A deductive qualitative content analysis methodology was applied to analyse selected research papers [28].

A Population, Interventions, Comparators, Outcomes, Study (PICOS) question design was used to guide the development of the review question [29]. The review question was for vulnerable populations: What nursing and midwifery governance and workforce interventions have increased access to quality health care at the primary health care level? The review aimed to source studies of nursing and midwifery interventions designed to increase access to health care with demonstrable outcomes for access and health equity. We sought to identify efforts to increase the supply of nurses and midwives, expand their roles and improve their regulation, performance management and remuneration, as well as opportunities for education and training and collaboration. Outcomes of interest included the following: improved availability of health services and accessibility, acceptability and quality of health care and enhanced health outcomes. Observational studies and quasi-experimental and non-experimental descriptive studies were considered suitable for inclusion, and a systematic search of the contemporaneous primary research literature published from 2005 to 2015 was undertaken. Electronic databases and the internet were searched using the keywords “nurs*” OR “nurse-led” OR “midwife*” OR “midwife-led” AND “leadership” OR “workforce” OR “staffing” AND “universal health coverage” OR “equity” OR “access”. The Medical Subject Headings (MeSH) headings “manpower” and “Nursing” and “Midwifery” and “Nurse Midwives” were also used and augmented by the keywords “equity” and “access”.

In this review, we adopted the World Health Organization’s (WHO) definition of primary health care (PHC) as the first level of contact that individuals, the family and the community have with the national health system, which constitutes the first element of a continuing health care process [30]. For the purpose of this paper, we defined a vulnerable population as a subgroup or subpopulation who, because of shared social characteristics, is at a higher risk of factors such as unsafe sex, stress, drug misuse and malnutrition that can impact upon health issues including HIV and diabetes as well as access to health care [31]. Examples of vulnerable populations include refugees, the elderly, the homeless, the poor, ethnic minorities, incarcerated people, children, those living in rural and remote settings, families experiencing domestic violence and adolescent mothers. Such groups may have multiple vulnerabilities and health risks.

**Fig. 2:**
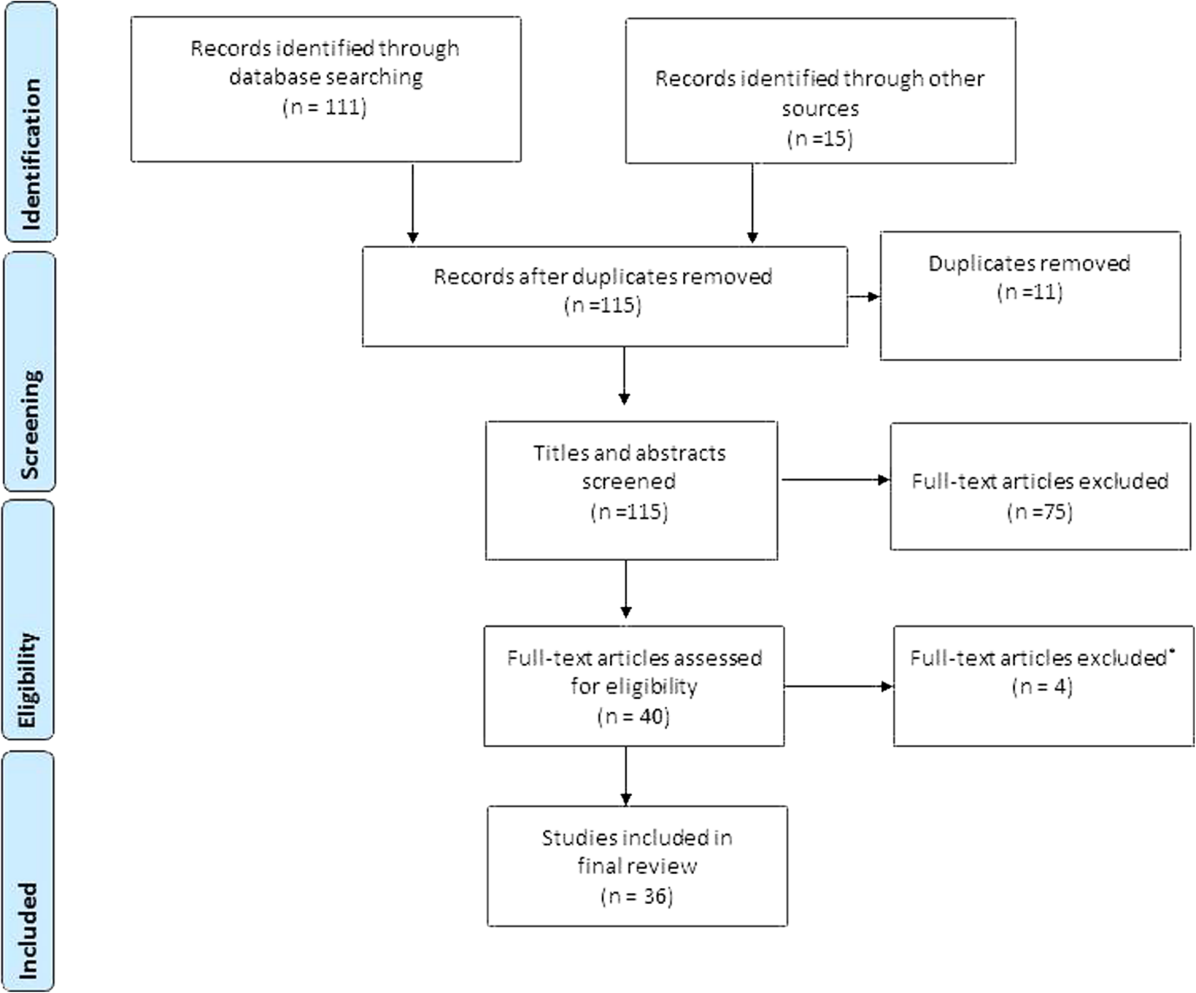
Overview of the literature review process

Retrieved records were first screened for their focus as per the review question by the first author and duplicates removed. As per the inclusion/exclusion criteria (see Table 1), discursive papers, those older than 10 years or whose focus was outside of the aim were removed. The Preferred Reporting Items for Systematic Reviews and Meta-Analyses (PRISMA) guidelines were used to report the review process [32] (see Fig. 2). The sources and numbers of papers retrieved and screened according to their relevance are outlined at Table 2. Seventy-five papers were then excluded at closer inspection as they were not concerned with interventions at the PHC level or focused on addressing the needs of vulnerable populations, nursing and/or midwifery workforce interventions were not described or they were discursive papers. Forty papers were appraised using the Critical Appraisal Skills Programme (CASP) tool for qualitative research [33], and Pluye et al.’s [34] scoring system was used to assess the non-experimental and mixed method studies. Four items were discarded due to methodological concerns [35–38].

**Table 1:** The inclusion/exclusion criteria applied to the screening of papers for the review

Included	Excluded
Primary health care	Hospital-based care
Nurse/midwifery-led health delivery	Care delivered by doctors, community or lay health workers
Study participants: vulnerable population groups	Study participants: general population, high socio-economic index
Interventions included: nurse/midwife education/training and/or increase in supply and/or human resource management (HRM) strategy and/or policy/ practice change and/or collaborative partnership arrangements	Interventions did *not* include: nurse/midwife education/training and/or increase in supply and/or HRM strategy and/or policy/ practice change and/or collaborative partnership arrangements
Outcomes included improvement in: acceptability/satisfaction/uptake of services and/or service quality and/or health outcomes and/or nurse/midwife capacity to promote, care and manage health issues	Outcomes did *not* include improvement in: acceptability/satisfaction/uptake of services and/or service quality and/or health outcomes and/or nurse/midwife capacity to promote, care and manage health issues
Research	Discursive or descriptive outlines of projects
English	Non-English
>2005	<2005

**Table 2:** Sources of retrieved and included papers

Source	Retrieved	Included
CINAHL	146	6
MEDLINE	136	12
PubMed	319	21
Scopus (Elsevier)	166	10
Current Contents Connect	49	15
Web of Science	24	4
ProQuest Health & Medicine	242	23
ScienceDirect	43	5
Google Scholar	57	9
*Total*	1182	111

Data were extracted from the 36 papers and first described according to general study characteristics (e.g. primary author, year of publication, context of the study), participants (age, gender and socio-demographic data), study aim, study design and findings (see Table 3). The conceptual framework outlined in Fig. 1 was then applied to identify the workforce, leadership and governance strategies described in the studies that had impacted on universal health access for vulnerable populations. A content analysis of the extracted text relating to each identified workforce, leadership and governance strategy was performed. This involved coding text according to emergent descriptions and then labelling and grouping them according to key HRH performance areas relating to policy, management, collaboration and education and training. Tables and concept maps were used to plot patterns and relationships across the categories and robustness assessed through critical reflection and discussion among the three authors.

**Table 3:** Summary of papers included in the review

Reference	Context	Method	Sample/participants	Aim	Findings
(Ayerle, Makowsky and Schücking 2012) [50]	Germany, home visiting	Mixed method: review of family records, 30 questionnaire and interviews with 14 mothers and 6 social workers	734 families whose vulnerability was scored according to factors such as conflict, drug abuse, social support, mental health, stress, teenage pregnancy, immigrant status	To examine the role of family midwives in the provision of care to vulnerable women and prevention of child neglect and abuse	The highest frequencies of care activities related to infant care and nutrition, giving advice on the mother-child relationship, and psychosocial support. The youth welfare services were significant collaborators. Mothers felt it was important to have early access to the FM and easy between-visits communication via phone calls or text messages. They appreciated the physical and psychosocial care for the infant and themselves.
(Bray et al. 2005) [51]	USA Eastern Carolina, clinics	Intervention study	160 minority African American patients in rural areas	To explore the efficacy of combining nurse-led-care management and interdisciplinary group visits for patients with diabetes mellitus	In the intervention group, 61% of patients had a reduction in HbA1c, and the percentage of patients with a HbA1c of less than 7% improved from 32% to 45% (P, 0.5).
(Chang et al. 2009) [39]	Kampala Uganda, community	Retrospective cohort study	360 urban slum dwelling HIV + people on ART of low socio-economic status in an already poor country and a significant proportion consists of displaced persons from the civil war in northern Uganda	To assess an alternative model, community-based, comprehensive antiretroviral programme staffed primarily by peer health workers and nurses	258 (72%) were active and on therapy approximately 2 years later.
(Chetty and Hoque 2013) [40]	South Africa: KwaZulu-Natal community psychiatric clinic	Quasi-experimental, non-equivalent, control group design study design	30 depressed Indian poor urban participants	To determine the effectiveness of a nurse-facilitated-cognitive-group intervention as an adjunct to antidepressant medication	At 6 weeks of the intervention, there was a decrease in the Beck Depression Inventory (BDI) scores of the intervention group and an increase in the BDI scores in the control group. At 12 weeks of the group intervention, the BDI scores for the intervention group showed a considerable reduction in their levels of depression, while the participants of the control group had a further increase in their scores—statistically significant difference between the groups, (*P* < 0.001)
(Coddington et al. 2011) [52]	USA Indiana, nurse-managed clinics	Non-experimental design review of child records	500 charts of patients from uninsured families or families on Medicaid	To assimilate evidence regarding quality of care received at nurse-managed clinics	Nurse-managed clinics met or exceeded national Healthcare Effectiveness and Data Information Set quality indicators as well as targets set by the Office of Medicaid Policy and Planning
(Dorney-Smith 2011) [53]	Hostel in South London, UK	Descriptive study using patient records to assess care usage and health outcomes	34 homeless males and females average age was 39 years	To assess outcomes of 1 year to reduce mortality and morbidity and secondary care usage at the hostel	34 hostel clients directly benefited from intermediate care. At the end of the year, the number of hospital admissions to the hostel had dropped 77% relative to 2008, and the number of accident and emergency (A&E) attendances had dropped 52%. Hospital “did not attends” (DNAs) were 22% lower. An economic evaluation found that the pilot project was cost neutral overall, and there is some evidence that health outcomes improved. intermediate care pilot project
(Ersser et al. 2013) [54]	Inner city metropolitan borough, England, UK GP clinics	Descriptive quantitative pretest-post-test design using health-related quality of life measures, severity measures, parental measure. Qualitative focus groups with parents	Families from high mobility, ethnic diversity and social deprivation setting	To evaluate the nurse-led Eczema Education Programme	Statistically significant impacts were observed on infant quality of life (*P* < 0.001), child quality of life (*P* = 0.027), disease severity (*P* < 0.001) and parental self-efficacy (*P* < 0.001). Improvements in child quality of life, parental efficacy and service impact were also evident from qualitative data. The cumulative total of all GP visits for selected participants post-EEP reduced by 62%.
(Frankenberg et al. 2009) [41]	Indonesia 13 of 26 provinces at village level	Secondary analysis of Indonesia Family Life Survey dataset (1993,1997, 2000) using logistic modelling	7224 households including the poor with low education in rural areas	To investigate the impact of scale up of midwives in access to midwifery services on women’s use of antenatal care and delivery assistance	Regardless of a woman’s educational level, the placement of village midwives in communities is associated with significant increases in women’s receipt of iron tablets and in their choices about care during delivery—changes that reflect their moving away from reliance on traditional birth attendants. For women with relatively low levels of education, the presence of village midwives has the additional benefit of increasing use of antenatal care during the first trimester of pregnancy.
(Gill et al. 2008) [42]	Rural KwaZulu-Natal in South Africa outreach clinics	Quasi-experimental study using clinical records and	284 rural poor with diabetes	To evaluate a nurse-led diabetes protocol and education-based system	A total of 284 patients were enrolled, with 197 followed for 18 months (13 died and 26% lapsed during the period). HbA1c was 11.6 ± 4.5% (sd) at baseline, 8.7 ± 2.3% at 6 months and 7.7 ± 2.0% at 18 months. There was a small associated increase in weight but no increase in hypoglycaemia. Subgroup analysis showed that education alone, without drug type or dose changes, also improved control (HbA1c 10.6 ± 4.2% baseline and 7.6 ± 2.3% at 18 months). The service was very popular with patients, families and other health workers.
(Goodman et al. 2005) [55]	England shire and inner London in homes and in care facilities	Focus groups and survey conducted in two settings with staff	74 community-based nurses and care home managers and staff providing care to older people in care homes and in their own home	To examine partnership of district nurses and care home staff providing care for older people	Nurses were the most frequent NHS professional visiting care homes. Although care home managers and district nurses believed that they had a good working relationship, they had differing expectations of what the nursing contribution should be and how personal and nursing care were defined. This influenced the range of services that older people had access to and the amount of training and support care home staff received from district nurses and the extent to which they were able to develop collaborative and reciprocal patterns of working.
(Griffiths et al. 2009) [56]	Australia: Western Sydney, community	Cross-sectional survey design, administered at two time points	327 women in a socially and economically disadvantaged community	To measure the effect of a community capacity-building programme implemented by Women’s Health Nurses	There was a significant improvement in mental health indicators and fewer women believed their physical or emotional problems imposed a considerable burden on their daily activities. They also believed people from other cultures were more likely to be accepted by neighbours and reported increased involvement in community activities as a direct result of the Villawood Icebreakers Project.
(Gross et al. 2010) [43]	Kenya	Secondary analysis of dataset from the Kenya Health Workforce Informatics System using logistic modelling	Nurses employed in Kenya’s public sector in rural and underserved areas	To analyse the effect of Kenya’s Emergency Hiring Plan for nurses on their inequitable distribution in rural and underserved areas	Of the 18 181 nurses employed in Kenya’s public sector in 2009, 1836 (10%) had been recruited since 2005 through the Emergency Hiring Plan. Nursing staff increased by 7% in hospitals, 13% in health centres and 15% in dispensaries. North Eastern province, which includes some of the most remote areas, benefited most: the number of nurses per 100 000 population increased by 37%. The next greatest increase was in Nyanza province, which has the highest prevalence of HIV infection in Kenya. Emergency Hiring Plan nurses enabled the number of functioning public health facilities to increase by 29%. By February 2010, 94% of the nurses hired under pre-recruitment absorption agreements had entered the civil service.
(Hesselink and Harting 2011) [57]	Netherlands in parent-child centres	Mixed methods: multiple case study field notes, observations and recordings of group classes, attendance logs, semi-structured individual interviews, a focus group interview, and structured questionnaires	119 first- and second-generation pregnant ethnic Turkish women with low education and minimal knowledge of Dutch	To evaluate a multiple risk factor perinatal programme ethnic Turkish community health workers in collaboration with midwives and physiotherapists	Most participants (82%) were first-generation ethnic Turkish, 47% had a low educational level, 43% were pregnant with their first child and 34% had a minimal knowledge of the Dutch language. The community health workers’ Turkish background was vital in overcoming cultural and language barriers and creating a confidential atmosphere. Participants, midwives and health workers were positive about the programme. Midwives also observed improvements of knowledge and self-confidence among the participants. The integration of the community health workers into midwifery practices was crucial for a successful programme implementation.
(Homer et al. 2012) [58]	Australia: Sydney in women’s homes, community health centre	Mixed methods: a focus group discussion and review of patient records	353 Aboriginal and Torres Strait Islander women who gave birth in 2007 and 2008	To examine the perspectives of women accessed the service	353 women gave birth through the Malabar service during 2007 and 2008. Over 40% of the babies born were identified as Aboriginal and Torres Strait Islander. Almost all the women had their first antenatal visit before 20 weeks of pregnancy. The service was successful in reducing the number of women smoking cigarettes during pregnancy. Women felt the service provided ease of access, continuity of care and caregiver, trust and trusting relationships.
(Hurley et al. 2013) [59]	Police custody suites in Tayside Scotland, UK	Qualitative study using focus groups and interviews underpinned by realistic evaluation method	28 nurses and nurse manager, police and security personnel caring for 4953 offenders	To explore the impact of nurses assuming leadership roles in delivering primary health care to detainees	The quality of clinical care for detainees improved, policing concerns for detainee safety were mitigated and forensic medical examiners were able to expand their specialist roles. Key supporting mechanisms in achieving these outcomes included generating collaborative practices, enacting clinical leadership and providing a forensic nursing educational programme to empower nurses to generate service provision and grow professional autonomy.
(Jackson et al. 2009) [60]	UK: North England, community health	Mixed methods: 21 survey and 9 focus group discussions at baseline, 6 and 12 months post-implementation	Individuals holding strategic posts in the Public Health and Nursing Directorates, health visitors, school nurses, voluntary sector staff delivering interventions to address health inequalities	To evaluate the community health team working in a defined geographical or topic area	Six themes emerged from the focus group data that illustrated key issues for the implementation of the CHT: “agreeing the focus”, “strong leadership”, “the challenge of communication”, “managing workloads and new ways of working”, “success of the CHT” and “outside influences”. Communication and heavy workloads were identified as key barriers to the success of the CHT in the questionnaire data.
(Labhardt et al. 2011) [44]	Primary health care clinics in rural districts in Central Cameroon	Open-label, three-arm, cluster-randomized trial in nurse-led facilities	221 poor, rural patients with hypertension and diabetes	To compare the effects of low-level facility-based interventions on patient retention rates for cardiovascular (CV) disease in an environment of task shifting and nurse-led care in	A total of 33 centres and 221 patients were included. After 1 year, 109 patients (49.3%) remained in the programme. Retention rates in groups 2 and 3 were 60% and 65%, respectively, against 29% in the control group. The differences between the intervention groups and the control group were significant (*P* < 0.001), but differences between the two intervention groups were not (*P* = 0.719). There were no significant differences in BP or fasting plasma glucose trends between retained patients in the study groups. Average monthly cost to patients for antihypertensive medication was €1.1 ± 0.9 and for diabetics €1.2 ± 1.1. Transport costs to the centres were on average €1.1 ± 1.0 for hypertensive patients and €1.1 ± 1.6 for patients with diabetes.
(Lamers et al. 2010) [61]	General practices, Limburg, the Netherlands	Randomized controlled trial	187 elderly patients with chronic obstructive pulmonary disease and symptoms of depression	To evaluate the effectiveness of a nurse-led minimal psychological intervention (MPI) in reducing depression and anxiety and improving disease-specific quality of life	Patients receiving the MPI had significantly fewer depressive symptoms (mean BDI difference 2.92, *P* = 0.04) and fewer symptoms of anxiety (mean SCL difference 3.69, *P* = 0.003) at 9 months than patients receiving usual care. Further, mean SGRQ scores were significantly more favourable in the intervention group than in the control group after nine months (mean SGRQ difference 7.94, *P* = 0.004).
(Larson et al. 2010) [62]	Rural general practices, Western Australia	Prospective cohort with before-after measures	83 patients from rural areas with asthma	Trialled the outcome for asthma patients of a brief, nurse-led, patient-education session with general practice review of an asthma action plan	Mean asthma control score decreased but did not reach statistical significance (*P* = 0.124). Quality of life improved for adults (Wilcoxon rank signed test for two related samples *P* < 0.001). The proportion of patients who had one or more unscheduled visits to their general practitioner over 12 months decreased from 23% to 13% (*P* = 0.178), and emergency department presentations decreased from 9% to 4% (*P* = 0.102).
(Leipert et al. 2011) [63]	Nurse practitioner services, rural southwest Ontario, Canada	Qualitative in-depth, face-to-face interviews using interpretive description methodology	9 rural women, aged 18–80 using nurse practitioner services	To explore rural women’s experiences with primary health care nurse practitioners	The participants in the study particularly appreciated the nursing knowledge of the NP, the time the NPs spent with them, and the thoroughness of the care provided by NPs. These foundational elements of the participants’ experiences with rural NPs created a sense of trust and respect, which lead to a collaborative partnership between the NP and the rural women.
(Markle-Reid, Browne and Gafni 2013) [64]	Home visiting Southern Ontario, Canada	3 randomized controlled trials using (HRQOL) (SF-36) and Health and Social Services Utilization Inventory from baseline to the end of the intervention	498 frail older adults with chronic health conditions and depression	To explore the main lessons learned from three trials to inform the design of best practice models for nurse-led health promotion interventions	Nurse-led HPDP interventions led to greater improvements in HRQOL compared with usual home care services. The cost analysis showed that even when the costs of the HPDP interventions were included in the total cost, there was no difference in the total per-person cost of health services between the HPDP intervention and usual home care.
(Markle-Reid et al. 2014) [65]	Home visiting Ontario, Canada	Prospective one-group pre-test/post-test study design. CES-D score, GAD-7, HRQOL (SF-12v2) and HSSUI. Thematic analysis of RN and PSW focus groups, content analysis of clients’ responses to the open questions	142 elderly home care clients	To examine the feasibility and acceptability of a new 6-month interprofessional nurse-led mental health promotion intervention and to explore its effects on reducing depressive symptoms	Of the 142 participants, 56% had clinically significant depressive symptoms, with 38% having moderate to severe symptoms. The intervention was feasible and acceptable to older home care clients with depressive symptoms. It was effective in reducing depressive symptoms and improving HRQOL at 6-month follow-up, with small additional improvements 6 months after the intervention. The intervention also reduced anxiety at 1 year follow-up. Significant reductions were observed in the use of hospitalization, ambulance services and emergency room visits over the study period.
(Mills 2014) [66]	HM Prison Risley, in the north west of England, UK	A retrospective audit of health records	27 male prisoners	To examine whether providing a nurse-led specialist diabetes service within the prison setting can improve the management of diabetes by reducing HbA1c	The results showed that hospital admission rates reduced, with only two admissions in 12 months. One was due to hypoglycaemia (overdose) and one due to infection. There were no admissions for diabetic ketoacidosis. Baseline HbA1c was 74 mmol/mol (8.9%); range 39–108 mmol/mol (5.7–12.0%). At 1-year follow-up, HbA1c had decreased to 58 mmol/mol (7.5%); range 56–119 mmol/mol (7.3–13.0%). The number of episodes of severe hypoglycaemia in the preceding 12 months was greatly reduced from 17 to 1 (*P* < 0.001).
(Neff, Kinion and Cardina 2007) [67]	USA: north-eastern Ohio	Quantitative descriptive review of patient records	334 Native American patients	To describe the nursing interventions provided at an urban nurse-managed centre to urban Native Americans.	The majority were over 40 years of age, were female, were single, completed high school and were poor and uninsured, and many were unemployed. The most frequent health problems were related to pain, cardiovascular symptoms, dentition problems and respiratory illnesses. The most frequent nursing interventions were for surveillance of physical signs and symptoms.
(Oliver et al. 2014) [68]	USA	Quantitative from multiple data sets	Medicare: national social insurance programme serves a large population of elderly and disabled individuals. Medicaid: social health care programme for families and individuals with low income and limited resources	Examine for a statistically significant relationship between the level of APRN practice allowed and recent nationwide, state-level analyses of Medicare or Medicare-Medicaid beneficiaries	States with full practice of nurse practitioners have lower hospitalization rates in all examined groups and improved health outcomes in their communities.
(Price et al. 2011) [45]	Hlabisa District, in northern KwaZulu-Natal, South Africa	Single-centre, observational cohort study.	80 type 2 diabetic patients in rural Africa	To determine the long-term (4 years) glycaemic outcome of a structured nurse-led intervention programme	Patients were of mean plus or minus SD, age 56 plus or minus 11 years, 70% were female, BMI 31.5 plus or minus 7.2 kg/m super(2) and HbA sub(1)c 10.8 plus or minus 4.2%. HbA sub(1)c fell significantly to 8.1 plus or minus 2.2% at 6 months and 7.5 plus or minus 2.0% at 18 months. By 24 months, it had risen (8.4 plus or minus 2.3%), and at 4 years post-intervention, it was 9.7 plus or minus 4.0% (still significantly lower than baseline, *P* = 0.015). BMI rose significantly at 6 and 18 months but by 48 months was not significantly different from baseline.
(Riley and Crawford 2010) [69]	General practice setting, Hawkes Bay, New Zealand	Audit of health facility records	265 consultations carried out over an 18-month period. 75% with Maori/Pacific Island and NZDep96 quintile groups 4 and 5 children and their whanau/families	To describe the implementation of a nurse-led, child-specific clinic to improve health outcomes for high needs	An outcome audit after 18 months demonstrated a significant (>30%) reduction in eczema severity, daily irritability and daily occurrence of pain. Post-intervention, fewer children were hospitalized and there was a 50% reduction in antibiotic use.
(Sears et al. 2008) [70]	Washington State, USA	Natural experiment was evaluated using descriptive techniques and a pre-post design	Rural workers 18–70 years of age who were injured in Washington and filed an accepted State Fund workers’ compensation claim between July 1, 2003, and June 30, 2005	(1) Describe the contribution by NPs to Washington’s workers’ compensation provider workforce, (2) evaluate change in provider availability attributable to SHB 1691 and (3) evaluate the effect of SHB 1691 on timely accident report filing.	NPs served injured workers with characteristics similar to those served by PCPs, but 22.0% of NPs were rural, compared with 17.3% of PCPs. Of claimants with NPs as their attending provider, 53.3% were injured in a rural county, compared with 24.7% for those with PCP attending providers. The number of NPs participating in the workers’ compensation system rose after SHB 1691 implementation, more so in rural areas. SHB 1691 implementation was associated with a 16-percentage-point improvement in timely accident report filing by NPs in both rural and urban areas.
(Schnippel et al. 2015) [49]	Van-based mobile clinic in two rural districts in South Africa	Service cost analysis	PHC to 2370 poor rural women who might access the service over a 12-month period	To evaluate the cost of service delivery	Fixed costs accounted for most of the total annual costs of the mobile clinics (85% and 94% for the two districts); the largest contributor to annual fixed costs was staff salaries. Average costs per patient were driven by the total number of patients seen, at $46.09 and $76.03 for the two districts. Variable costs for Pap smears were higher than for other services provided, and some services, such as breast exams and STI and tuberculosis symptoms screening, had no marginal cost.
(Shumbusho et al. 2009) [47]	Three rural primary health centres in Rwanda	Review of medical records from September 2005 to March 2008	1076 rural patients enrolled in HIV care and treatment services	To evaluate a nurse-centred antiretroviral treatment programme	Of the 435 patients who initiated ART, the vast majority had adherence and side effects assessed at each clinic visit (89% and 84%, respectively). By March 2008, 390 (90%) patients were alive on ART, 29 (7%) had died, one (1%) was lost to follow-up and none had stopped treatment. Patient retention was about 92% by 12 months and 91% by 24 months. Depending on the initial stage of the disease, mean CD4 cell count increased between 97 and 128 cells/ml in the first 6 months after treatment initiation and between 79 and 129 cells/ml from 6 to 24 months of treatment. Mean weight increased significantly in the first 6 months, between 1.8 and 4.3 kg, with no significant increases from 6 to 24 months.
(Small et al. 2008) [71]	Canada	Qualitative descriptive semi-structured qualitative interviews	50 individuals recruited from a cohort of Safe Injecting Facility (SIF) users	To investigate IDU perspectives regarding the impact of SIF on access to care and treatment of injection-related infections	Narratives indicate the availability of on-site nursing attention at the SIF-facilitated uptake of health services. IDU reported that the facility provided assessment and care of injection-related infections, as well as enhanced access to off-site medical services. The presence of professional nursing personnel within a sanctioned drug consumption setting serves to address social and structural barriers that often impede IDU access to health care.
(van Griensven et al. 2008) [48]	Two government-run health centres in Kigali, Rwanda	Programme treatment and outcome data from 2 facilities. Interviews with staff and MSF programme records were reviewed to describe the organization of the programme	315 children with HIV	To describe the nurse-centred paediatric ARV programme implemented in two government health centres with details of its psychosocial aspects and treatment outcomes	A total of 315 children (<15 years) were started on ARVs, at a median age of 7.2 years (range: 0.7–14.9). Sixty percent were in WHO clinical stage I/II, with a median CD 4% of 14%. Eighty-nine percent (*n* = 281) started a stavudine-containing regimen, mainly using the adult fixed-dose combination. The median follow-up time after ARV initiation was 2 years (interquartile range 1.2–2.6). Eighty-four percent (*n* = 265) of children were still on treatment in the programme. Thirty (9.5%) were transferred out, eight (2.6%) died and 12 (3.8%) were lost to follow-up. An important feature of the study was that viral loads were done at a median time period of 18 months after starting ARVs and were available for 87% of the children. Of the 174 samples, VL was <400 copies/ml in 82.8% (*n* = 144). Two children were started on second-line ARVs. Treatment was changed due to toxicity for 26 children (8.3%), mainly related to nevirapine.
(Welch et al. 2011) [72]	Urban community health centre, USA	Randomized controlled trial	46 poor Hispanic patients with type 2 diabetes	To evaluate the clinical usefulness of a nurse-led comprehensive diabetes care programme	Patients receiving the intervention (IC) had a significant improvement in A1C from baseline to 12-month follow-up compared with the control (AC) (−1.6 ± 1.4% versus −0.6 ± 1.1%; *P* = .01). The proportion of IC patients meeting clinical goals at follow-up tended to be higher than AC for A1c (IC = 45%; AC =28%), systolic blood pressure (IC = 55%; AC = 28%), eye screening (IC = 91%; AC = 78%) and foot screening, (IC = 86%; AC = 72%). Diabetes distress and treatment satisfaction also showed greater improvement for IC than AC (*P* = .05 and *P* = .06, respectively), with no differences for depression.
(Wetta-Hall 2007) [73]	USA: Sedgwick County Kansas	Cross-sectional study health records review and survey	492 low-income uninsured programme participants population	To assess the impact of a collaborative community case management programme on a low-income uninsured population	A statistically significant (48%) reduction in total ED visits resulted in an estimated charge avoidance of US$ 1 446 280. Physical health status improved significantly; however, mental health status and health locus of control scores showed minimal change.
(Wray, Walker and Fell 2008) [74]	UK: Hull	Quantitative descriptive design using a survey	160 nursing and midwifery university students	To examine student attitudes prior to and on completion of a module on social inclusion/exclusion	The data demonstrated that the majority of students surveyed held views that were generally positive and inclusive. Yet, a small group of respondents held stereotypical views potentially compromising their ability to provide health care.
(Yates et al. 2010) [49]	Snake Park clinic in Meserani, Tanzania	Prospective study of health facility data between April 2007 and the end of 2009, received treatment for snakebite envenomation	85 patients rural poor patients	To examine the management of snakebites by the staff of a rural clinic:	The 85 snakebite cases had a mean age of 23 years and a male:female ratio of 1.4. In some cases, the seeking of treatment from traditional healers delayed treatment at the clinic. After being bitten, the snakebite cases travelled a mean of 82 km (range = 2–550 km) to reach the clinic. Thirty-two (37%) cases were unable to identify the snake that had bit them. Forty-two of the snakebite cases received antivenom. Only one patient (1%), a 12-year-old girl, died as the result of a snakebite, but another six (7%) each required a skin graft or the amputation of a limb or digit.

## Results

Thirty-six papers were included in the review. Eleven papers described research in low- and middle-income country (LMIC) contexts (Uganda, South Africa, Indonesia, Kenya, Cameroon, Rwanda and Tanzania) [39–49] and 25 from high-income contexts (HIC) (Germany, the United Kingdom, the United States, Canada, Australia, The Netherlands, New Zealand) [50–74]. Four papers were concerned with outcomes related to midwife-led interventions (antenatal, intrapartum and postnatal care and prevention of child abuse) [41, 50, 57, 58], while 32 focus on nursing, some on specific health issues including asthma [62], eczema [54, 69], cardiovascular disease [44], diabetes care [42, 45, 51, 66, 72], HIV/AIDS [39, 47, 48], mental health [40, 61, 65] and women’s health [46].

Different types of nurses were described providing care at the first point of contact in the various studies. These nurses included the following: nurse practitioners [63, 68, 70] and advanced nurse practitioners [52, 65, 67], certified nurse specialist [51], psychiatric nurses [40], paediatric nurses [52] intermediate care nurses [53], community nurses [64, 65] community-based nurses [54, 55], community staff nurses [44, 55], district nurse [55], specialist dermatology nurses [54] or practice nurses working with general practice (GP) doctors [54, 62, 69], diabetes specialist nurses [42, 66], diabetes nurses [42, 72], local diabetes trained nurse [45], women’s health nurses [56], public sector nurses [43], registered nurses [43, 44, 59, 64, 65, 67, 73], registered nurses working in a community nursing agency [64], registered nurses working in a public health agency [64], nurse managers [59], respiratory nurse educators [62], nurse care managers [65], mental health nurses [65], PHC nurses [42, 45, 46], child health nurses [69], auxiliary nurses [40], enrolled nurses [40, 43], nurse assistants [44], general nurses [40], primary care nurses [60], school nurses, [60], public health nurse facilitators [60], head nurses [44], antiretroviral (ARV) nurses [48], student nurses [74].

Specific vulnerable populations formed the focus of many papers including children [48, 50], the elderly [55, 61, 64, 65], ethnic minorities [40, 51, 57, 72], indigenous people [58, 67], the homeless [53], the poor [39, 41, 44, 52, 54, 56, 60, 68, 69, 73], prisoners [59, 66], those living in rural locations [42, 43, 45–47, 49, 62, 63, 70] and people with substance use disorders [71]. Some populations had multiple vulnerabilities such as families described in the Ayerle et al. paper [50]. Most studies were quantitative [39–47, 49, 51–53, 56, 61, 62, 64, 66–70, 72–74], followed by mixed methods [48, 50, 54, 57, 58, 60, 65] and qualitative [59, 63, 71] research. Most studies were intervention studies where a new or existing nurse or midwife-led initiative was evaluated, while other research examined the effect of a service where the nurse or midwife had played an important role. Other studies analysed in this review involved the secondary analysis of data to examine the impact of nursing or midwifery care over time [41, 43, 52]. One study focused on an education intervention to prepare student nurses to care for vulnerable populations [74]

Table 4 outlines the focus of policy, governance and workforce strategies and approaches described in the studies as part of outlined interventions to improve the supply or support of midwives and nurses to deliver care to enhance the health outcomes of vulnerable groups. Twenty-seven of the 36 papers contained some mention of human resource management (HRM) strategies and approaches, while 22 papers described collaboration, 14 education and training efforts and 11 leadership and governance approaches. Only three papers contained descriptions of policy, HRM strategies, collaboration and education and training efforts [41, 48, 59]. The content analysis of the papers provided further detail and insight into how such strategies and approaches had been utilized to improve access to the care delivered by nurses and midwives. These findings are outlined below according to the key areas.

**Table 4:** Area of strategies described in the studies included in the review

	Leadership/governance		Workforce	
Reference	Policy and practice	HRM	Education and training	Collaboration
(Ayerle, Makowsky and Schücking 2012) [50]		✓		✓
(Bray et al. 2005) [50]		✓		✓
(Chang et al. 2009) [39]		✓	✓	✓
(Chetty and Hoque 2013) [40]		✓		
(Coddington et al. 2011) [52]		✓		✓
(Dorney-Smith 2011) [53]		✓	✓	✓
(Ersser et al. 2013) [54]			✓	
(Frankenberg et al. 2009) [41]	✓	✓	✓	✓
(Gill et al. 2008) [42]		✓	✓	
(Goodman et al. 2005) [55]		✓		✓
(Griffiths et al. 2009) [56]		✓		✓
(Gross et al. 2010) [43]	✓	✓		
(Hesselink and Harting 2011) [57]				✓
(Homer et al. 2012) [58]				✓
(Hurley et al. 2013) [59]	✓	✓	✓	✓
(Jackson et al. 2009) [60]	✓			✓
(Labhardt et al. 2011) [44]		✓	✓	
(Lamers et al. 2010) [61]		✓	✓	
(Larson et al. 2010) [62]			✓	✓
(Leipert et al. 2011) [63]	✓	✓		✓
(Markle-Reid, Browne and Gafni 2013) [64]				✓
(Markle-Reid et al. 2014) [65]		✓	✓	✓
(Mills 2014) [66]				✓
(Neff, Kinion and Cardina 2007) [67]	✓	✓		✓
(Oliver et al. 2014) [68]	✓	✓		
(Price et al. 2011) [45]		✓	✓	
(Riley and Crawford 2010) [69]	✓	✓		
(Sears et al. 2008) [70]	✓	✓		
(Schnippel et al. 2015) [49]		✓		✓
(Shumbusho et al. 2009) [47]	✓	✓	✓	
(Small et al. 2008) [71]		✓		
(van Griensven et al. 2008) [48]	✓	✓	✓	✓
(Welch et al. 2011) [72]		✓		✓
(Wetta-Hall 2007) [73]				✓
(Wray, Walker and Fell 2008) [74]			✓	
(Yates et al. 2010) [49]		✓		

### Leadership and governance

The analysis of 11 papers identified policies at the national and state level that had impacted upon the increased supply and coverage of nursing and midwifery staff and scope of practice. Two papers describe the Indonesian and Kenyan government’s efforts to increase the numbers of midwives and nurses through scaling up education and training and deployment to underserved areas, particularly poor rural communities. In Indonesia, the presence of village midwives led to significant increases in women’s acceptance of iron tablets and use of antenatal care during the first trimester of pregnancy [41]. In Kenya, the Emergency Hiring Plan (EHP) increased the number of nurses in remotes areas by 37% and enabled the number of functioning public health facilities to increase by 29% [43]. Two papers from Rwanda [47, 48] describe the Ministry of Health’s collaboration with international donors to develop models of task shifting, or the delegation of specific HIV/AIDS tasks in a context of chronic workforce shortages, to optimize nurse performance to deliver care to adults and children with HIV/AIDS. The government’s commitment also included the provision of additional nursing staff supported by laboratory staff and ongoing laboratory services, training and antiretroviral procurement [48].

Other policy and regulation efforts to expand nurses’ scope of practice to better deliver care to vulnerable populations are described in two papers with respect to nurse practitioners (NPs) and advance nurse practitioners (ANPs). Leipert et al. [63] refer to the Canadian Nurses Association and Government-supported initiative to better integrate NPs into health care while a study by Oliver and others demonstrates that American States with legislation allowing full practice of nurse practitioners have lower hospitalization rates among Medicare-Medicaid beneficiaries and improved health outcomes in their communities [68]. Research by Sears et al. also found that the implementation of the Washington State workers’ compensation system, which involved the expansion of the NP scope of practice to increase access to health care for injured workers in rural areas, resulted in increased NP participation and a 16-percentage-point improvement in timely accident report filing by NPs particularly in rural areas [70].

Government support for the introduction and expansion of nurse-led clinics through policy and funding investment was reported in a New Zealand study whose examination of consultations over an 18-month period demonstrated a significant (>30%) reduction in child eczema severity, fewer child hospitalisations and a 50% reduction in family antibiotic use among Maori/Pacific Island and other low socio-economic groups [69]. A review of patient records at one of the first urban academic-nurse-managed clinics in America reported a range of interventions delivered to 334 urban Native Americans [67]. This nurse-led care, according to a previous study of this service, was valued by Native Americans particularly the advocacy that nurses provided on behalf of clients and the continuity of care [75].

Other papers provide examples of nursing leadership in practice. Leadership was reported to underpin successful collaboration between police and nurses in a local government region in Scotland that allowed nurses to focus on the delivery of quality care for prisoners [75]. “Strong leadership” from the Public Health Nurse Facilitator was regarded as critical to the functionality of the community health team in England to provide mainly nursing care to deprived areas [60].

### Nursing and midwifery management

Descriptions of the *roles* of nurses and midwives featured in the reviewed papers, followed by issues of staffing supply, distribution and skills mix; workload; supervision; performance management; and remuneration, financial incentives and staffing costs. A wide range of primary health care nursing and midwifery roles was described in the papers that contributed to improved outcomes for vulnerable populations. These included taking patient histories, screening of new patients, the surveillance of physical signs and symptoms, conducting diagnostic tests and screening for side effects, cleaning wounds, infection control, prescribing and provision of medication, assessment of social issues, education and counselling of patients, establishing contracts with patients to manage health issues, completing and submitting official forms and reports, referral and community health promotion and advocacy. Nurses and midwives worked in clinics and provided outreach services and home visiting. NP roles were described as autonomous [63] and licensed as independent health care providers whose roles were similar to those of primary care physicians [70] and who consulted with doctors as needed [63]. However, one study noted that where NPs required a doctor’s signature to process compensation for injured workers, delays in health care and accident report filing were observed, particularly for rural or underserved populations [70].

Role challenges and issues were noted in two studies. Proscribed and narrowly defined nursing roles in two studies presented by Goodman et al. were said to result in nurses limiting their involvement in district nursing efforts which led to nurses feeling ambivalent about their roles [55]. Concerns were raised in a research by Dorney-Smith about role isolation and conflict that nurses may experience while working in a hostel for homeless people which may lead to “burn out”. A number of suggestions were offered to deal with such issues if they occurred, including the presence of management at a weekly handover meeting to provide supervision and support, monthly one-to-one and group supervision and rotations to enable time away from the hostel at the main team office [53].

Many of the studies outlined the role that nurses and midwives played in the delivery of new interventions which involved the addition or specialization of the tasks they performed. This was described as an expanded or extended role using delegation or task shifting. The design and implementation of community-based health promotion, education and advocacy were regarded as expanding the role of Women’s Health Nurses in a paper outlining a programme focused on building resilience and social capital through networks in a disadvantaged community in urban Australia [56]. The addition of the provision of care to injured workers as part of newly introduced compensation legislation was also described as expanding the role of NPs [70]. The roles of two diabetes nurses who set up a weekly diabetes clinic in a rural area of South Africa were seen to have taken on extended nursing roles while diabetes-related tasks were delegated to other nurses who showed interest in the clinic in order to optimize the service [45]. Shifting specific tasks to nurses, in addition to their usual work, to scale up the care of poor rural dwelling Africans with HIV/AIDS, hypertension and diabetes was the focus of research outlined in two papers [44, 47]. Supportive mechanisms were developed to assist these nurses in their role, including the modification of clinical data collection forms, checklists and job aides to guide the process of data collection [47] and clear processes for setting up contracts with patients for disease management [44]. Shifting tasks to nurses through nurse-led clinics was found to have positively impacted upon doctors’ workload [69].

Strategies to optimize nurse’s performance and ensure appropriate workloads through task shifting from nurses to other health workers were described in two papers included in the review. Health support workers were employed in a homeless hostel, and tasks associated with dressings, observations, and medications that would normally have been undertaken by nurses shifted to this cadre so that the caseload for the nurse could be increased to best serve clients [53]. In another study, nurses’ administrative work and data collection were shifted to receptionists, counselling work was shifted to counsellors and community support groups and lab staff took on blood collection [48]. In one study, nurses viewed the establishment of a community health team to enhance services to disadvantaged communities as additional work, particularly in the initial 12-month set-up phase [60]. One nurse in this study said she was concerned that this work was not part of her paid role, highlighting the need for tasks that address the needs of vulnerable groups to be clearly identified and integrated into nurses’ roles and appraised.

In another study, substantial increases in nurses’ workload associated with the fast scaling up of an antiretroviral therapy (ART) programme in Rwanda were introduced alongside a performance-based financing mechanism [48] where nurses were contracted and remunerated according to their performance. In this study, nurse retention was found to be high which contrasts with the approach taken in other research in Rwanda where nurses did not receive salary increases or other incentives for their new role under this task-shifting initiative [47]. Staffing costs can often be the largest component of a health service; however, another study in Africa found that the provision of multiple nurse-led services via a mobile cervical cancer screening programme was an approach to potentially expand access to health care to rural populations without added costs [46]. Non-financial incentives may therefore be just as important when it comes to delivering quality care. Nurse performance in South Africa has been associated with adequate training, mentoring and support [47]. The nurse’s performance in other studies in this review was supported through a range of supervisory and recording mechanisms. Checklists and reporting forms assisted nurses to adhere to guidelines in clinical protocols [46, 61, 65] while nurses were supervised by senior nurses [59] or their work reviewed by primary care physicians [47, 51, 62].

### Education and training

Education and training activities are described by a number of authors to support nurses and midwives to deliver care. In a study involving nurse-led care to prisoners, nurse education was aimed to enhance the nurses’ existing core capabilities and “minimize organizational risk while supporting nurses’ role expansion” [59]. In inner city English general practices, a cascade model was employed to train specialist nurse trainers to train and mentor 23 community-based practitioners to deliver an eczema education programme to disadvantaged families [54]. However, high nurse turnover and the high levels of dermatological expertise required to deliver the sessions confidently led to this model being discontinued and replaced with specialist nurse delivery [54]. In a South African diabetes programme, an English expert nurse was employed for the first 12 months of the project to train and support the newly appointed local diabetes nurse [42, 45]. Nurse training in the start-up phase of a nurse-led service was also noted in other studies [44, 65] along with regular continuing professional education provided by special nurses [62] or doctors [39, 61].

Details of physician involvement in nurse training are provided in the studies examining nurse-led ART care in Rwanda [47, 48]. In addition to formal training through the National HIV/AIDS programme that involved physician-observed practicums, three nurses received training by doctors at each PHC facility, as well as ongoing supervision and mentorship, before they were allowed to consult patients independently [47]. An additional nurse also received the training at each PHC facility, in order to ensure replacement of staff in the ART service in case of need.

One study in this review involved the pilot of a module on social inclusion/exclusion with a group of nursing and midwifery university students. The study found that the majority of students surveyed on completion of the module held views that were generally positive and inclusive. However, some respondents held stereotypical views potentially compromising their ability to provide health care to vulnerable groups suggesting that ongoing efforts to build empathy and commitment to addressing equity may be required throughout nursing and midwifery undergraduate education [74].

### Collaboration

This review identified significant collaborative efforts between nurses and midwives and other health providers and health care organizations, across the community, education and justice sectors, and with communities and consumers/clients/patients. Despite nurses leading the provision of services to vulnerable groups in many studies, they worked with doctors by reviewing patients together [48, 51, 52, 66] or referring when needed [53, 62, 72]. Papers described nurses also working closely with other nurses [45, 60] and midwives working with child and family health nurses [58]. In addition, nurses worked in teams with allied health professionals including physiotherapists, occupational therapists, social workers, dietitians and speech pathologists and non-professional personal support workers [64, 65], prison pharmacists [66], dieticians [72] and community support workers [45]. Communication between different professional groups was described as difficult in the early stages of the implementation of a community health team but improved over time when professionals became “aware of each other’s public health work, more confident in contacting each other to ask questions, and were beginning to share skills” [60]. Participants in this study reported that interprofessional communication would not have occurred had it not been for the community health team initiative [60].

Collaboration was also found to be central to the ability of nurses and midwives to deliver culturally competent care. In a New Zealand study, nurse communication with families and the acceptability of health care was enhanced through working with interpreter services and grounding their practice in Maori understandings of health [69]. In Australia, the staffing of The Malabar service by midwives who worked alongside Aboriginal Health Education Officers and community health workers helped to ensure community engagement, cultural safety and individualized continuity of care [58]. The importance of collaborating with community health workers to provide appropriate care to pregnant migrant Turkish women is noted in the study by Hesselink and Harting [57] where Dutch midwives and physiotherapists worked with ethnic Turkish community health workers. In addition to increasing the supply of midwives to scale up access in Indonesia, the study by Frankenberg, et al. also described the importance of midwives in developing collaborative relationships with traditional village midwives [41]. These efforts not only aimed to ensure referral to skilled providers but also acknowledged the important cultural role played by traditional birth attendants in supporting women before, during and after birth [41]. In an American study, bicultural/bilingual nurses were employed as part of a diabetes team to provide culturally acceptable care to poor Hispanic patients with type 2 diabetes [72].

The findings of two papers in the review noted the importance of collaboration with the public health sector at the national level and international donors to ensure that efforts to deliver nurse-led HIV/AIDS care are co-ordinated, well supported and contributed to health system strengthening efforts [47, 48]. Collaboration and strategic partnerships with area hospitals, community health centres and public health departments were also described in relation to the operation of a large nurse-managed clinic in America to provide ancillary and specialty health care services to the uninsured [67].

Collaboration and partnerships across sectors were also regarded as a necessary part of care provision. In Germany, The Youth Welfare Services were significant collaboration partners enabling midwives to develop trusting relationships with mothers and provide continuity of care [50]. In other studies, partnership with the voluntary sector [53], police [59] and social workers [73] not only improved the quality of care that vulnerable groups received but also assisted with linking to other services and advocacy efforts. Finally, collaboration with the community is described as a significant component of nursing and midwifery care. This includes nurses undertaking community needs assessments to inform the planning of nurse-managed care to urban Native Americans [67], consultations with community members to inform the development of activities to support networking and social cohesion [56] and working with women [63] and people living with HIV/AIDS who are patients themselves to ensure that all patients received home visits, medications and social support [47].

## Discussion

The transition from the Millennium Development Goals to the post-2015 Sustainable Development Goals (SDGs) has provided an opportune window to review heath goals. Global discussions and consultations on the SDGs have focused on the importance of interconnectedness of human development and, in health, on achieving universal health coverage with equity as a central principle. A critical element to deliver that agenda is having a workforce that can deliver accessible health care, including to the poorest, as well as to reach those who are most disadvantaged.

This literature review shows that nurses and midwives have and continue to contribute to providing universal access and reducing heath care disparities through the support of interventions in a number of domains. This paper has conceptualized these interventions using a framework that has highlighted the role of policy and regulation, leadership processes and practices, human resource management, education and training and collaborative partnerships. While in all countries nurses and midwives are currently working towards achieving universal access, most efforts remain undocumented in published literature. Of the 36 papers included, the majority were from high-income contexts with 11 from low- and middle-income countries, of which 10 were from African countries experiencing enormous human resources in health challenges. Only four papers related to midwife-led interventions indicating a gap in knowledge concerning evidence-based policy and workforce interventions in this context. While we were able to identify approaches to increase the number of well-trained, motivated nurses and midwives to provide the services to meet patients’ needs thereby increasing access to quality health care services, we were only able to locate one study that identified cost effective nursing care [46]. However, it is not clear if this mobile nursing health service was more affordable to women suffering financial hardship than other services.

The review shows that national and state policies to increase the supply, scope of practice and coverage of nurses and midwives to improve PHC access to address inequity were successful in both low- and high-income countries. Policy was also found to enable nurses to carry out expanded roles. Other authors have called for the need for nurses to pay close attention to the context of legislative and organizational changes in nurse regulation which may constrain nursing’s capacity to achieve health equity [76].

In our review, successful initiatives were accompanied by substantial long-term investments in infrastructure, training and improvement of working conditions of the health workforce. Such investment involves health system strengthening as part of efforts to increase universal health coverage which was evident in the context of several studies in this review [41, 43, 47, 48]. A recent study highlights the importance of equity as a measurable component of universal health coverage to ensure health care for vulnerable populations and summarizes useful indicators and frameworks [77]. This will include the ongoing assessment of midwifery and nursing services received by vulnerable groups [78]. However, the effective implementation of policies to increase access to health care requires the active participation of nurse leaders, particularly where the needs of unique populations must be addressed, to promote equity in nursing policy and practice [79, 80].

In terms of human resource management, in particular the roles and responsibilities of nurses and midwives, where staff took on expanded roles in their work, there was a positive impact in the delivery of health care. Successful expansion depended on clearly defining roles, providing additional training and supportive mechanisms for taking on these roles. In addition, incentives, both financial and non-financial including opportunities for further training and career development, were shown to be important factors for staff retention and performance that have been highlighted in other literature [81]. In resource-poor settings, the critical analysis of tasks performed by nurses and midwives led to some administrative tasks being assumed by other members of the health team, freeing up nurses and midwives to focus on delivering care especially to those with HIV/AIDS. The review findings concerning support for the expansion of nurses’ and midwives’ roles and the delegation of tasks to nurses from other cadres and from nurses to other staff concur with research into task shifting and sharing [82, 83].

The review found that in terms of collaboration, partnerships and communication that working as members of a health team was key in improving care delivery. Teams were found to work best when there was an understanding and appreciation of the roles and responsibilities among all members of the team, as well as staff with cultural competence and strong relationships with partners. Other literature, not included in this review, provides insight into fostering such collaborative practice through skills building and linkages. Connolly et al. outlines innovative nursing education in community-based health centres [84], and Sullivan demonstrates how partnerships with community agencies can increase the cultural competency of nurses [85]. These approaches add to the insight provided in the paper by Wray et al. [74] in this review and other reports [86–88] where social inclusion and cultural learning positively influenced student nurse clinical and research skills and attitudes. However, more research is required to examine changes in ethical practice and attitudes and the relationship with actual access and uptake of services [89].

The review has several limitations. Despite a structured search of a large number of databases, some studies may have been missed as our keywords may not have been sufficient to retrieve them. The use of a conceptual framework assisted in focusing the analysis, but detail may have been lost in an effort to produce a comprehensive synthesis of useful insights for nurses, midwives and decision makers.

## Conclusion

This review has synthesized literature documenting the interventions and approaches to support nurses and midwives to provide universal access to PHC for disadvantaged populations. While much is being done in many countries, few collect data or conduct operational research to document the challenges being faced or the strategies used to retain nurses and midwives to work with vulnerable populations. It is critical that nurses and midwives, especially in LMIC, be encouraged to document best practices and publish these so that knowledge can be shared. A conceptual framework for nursing and midwifery leadership and workforce interventions may be useful for not only documenting lessons learned but also designing, planning and evaluating these experiences and their impact on universal health access particularly for vulnerable populations. Such guidelines may help to focus efforts to reduce health inequity and achieve universal health coverage.
